# Baicalein antagonizes acute megakaryoblastic leukemia in vitro and in vivo by inducing cell cycle arrest

**DOI:** 10.1186/s13578-016-0084-8

**Published:** 2016-04-01

**Authors:** Chunjie Yu, Jiancheng Zeng, Zhenzhen Yan, Zi Ma, Shangqin Liu, Zan Huang

**Affiliations:** College of Life Sciences, Wuhan University, 16 Luo-Jia-Shan Road, Wuhan, 430072 Hubei People’s Republic of China; Department of Chinese Medicine, Zhongnan Hospital of Wuhan University, Wuhan, Hubei People’s Republic of China; Department of Hematology, Zhongnan Hospital of Wuhan University, 169 Donghu Road, Wuhan, 430071 Hubei People’s Republic of China

**Keywords:** AMKL, Baicalein, Proliferation, apoptosis, Cell cycle arrest, Differentiation

## Abstract

**Background:**

The prognosis of acute megakaryoblastic leukemia (AMKL) is really dismal, which urges for development of novel treatment. Baicalein is one type of flavonoids extracted from *Scutellaria baicalensis Georgi* (Huang Qin). It inhibited cell proliferation and subcutaneous tumor formation of many tumor cell lines. However, whether baicalein possesses anti-AMKL activities has not been tested.

**Results:**

We found that baicalein potently inhibited proliferation of multiple AMKL cells including CMK, CMY, Y10, 6133, and 6133 MPL/W515L due to apoptosis and cell cycle arrest at G1 phase. Unexpectedly, caspase inhibitor z-VAD-fmk did not restore cell proliferation. In contrast, ectopic expression of Cyclin D1 efficiently antagonized the inhibitory effect of baicalein. In addition, baicalein induced differentiation of 6133 MPL/W515L cells. Finally, baicalein promoted mice survival and reduced disease burden in a mouse model of AMKL.

**Conclusions:**

Baicalein possesses potent anti-AMKL activity in vitro and in vivo. Baicalein may be a potent reagent for AMKL therapy.

## Background

Acute megakaryoblastic leukemia (AMKL) is a rare subtype of acute myeloid leukemia classified as M7 by FAB [1–3]. Representing approximately 1 % of all leukemia during childhood, AMKL is the most common type of acute myeloid leukemia (AML) in young children with Down syndrome with an incidence of 0.5 per million per year [4]. GATA-1 mutations are tightly associated with AMKL in Down Syndrome children and sporadic mutations have found in adult AMKL [5, 6]. The only recurrent genetic alterations identified were *OTT*-*MAL* and *CBFA2T3*-*GLIS2* in children type of AMKL [7–9]. Although intensive multidrug chemotherapy has been employed, the prognosis of AMKL is really dismal with median survival time 40 weeks [10–12]. So far, no target therapy is available for AMKL. Recently, Aurora kinase A was proposed to be a therapeutic target for chemicals such as MLN8237 to promote polyploidization and differentiation in AMKL, shedding a light on target therapy of this fatal disease [13]. Nevertheless, it is still early to warrant a successful clinical result and the poor situation urges for the development of novel therapeutic methods.

Traditional Chinese herbs have been recognized as a good resource for drug development. Among them, baicalein is very attractive for its anti-inflammatory, anti-microbial, neuro-protective, and anti-cancer properties [14]. Baicalein is one type of flavonoids isolated from the dried root of *Scutellaria baicalensis Georgi* (Huang Qin). It has been reported to inhibit proliferation and induce apoptosis in various human cancer cell lines, such as liver, colon, breast, lung, myeloma, and pancreatic cancer cells [15–19]. Previous studies suggest baicalein and other two closely related flavonoids (wogonin and baicalin) may inhibit proliferation and induce apoptosis mainly through causing cell cycle arrest, modulating activities of some important signaling molecules including AKT, IκB-α,p53, and notch. [18, 20–22], promoting reactive oxygen species (ROS) product, releasing cytochrome c, regulating mitochondrial membrane potential, or activating caspase cascade [23–25]. Yet very few studies have been done in leukemic cells. Recently, wogonoside was reported to improve survival of NOD/SCID mice xenografted with AML blasts [26]. Thus these flavonoids may possess great potential for development of anti-leukemia drugs.

In the present study, we investigated the effects of baicalein on AMKL cells. We found that baicalein potently inhibited AMKL cell proliferation in vitro by inducing cell cycle arrest. In vivo, baicalein reduced disease burden and promoted mouse survival in an AMKL mouse model. Our study identified baicalein as a potent chemical compound that may be beneficial for AMKL therapy.

## Results

### Baicalein potently inhibits proliferation of AMKL cells

**Fig. 1:**
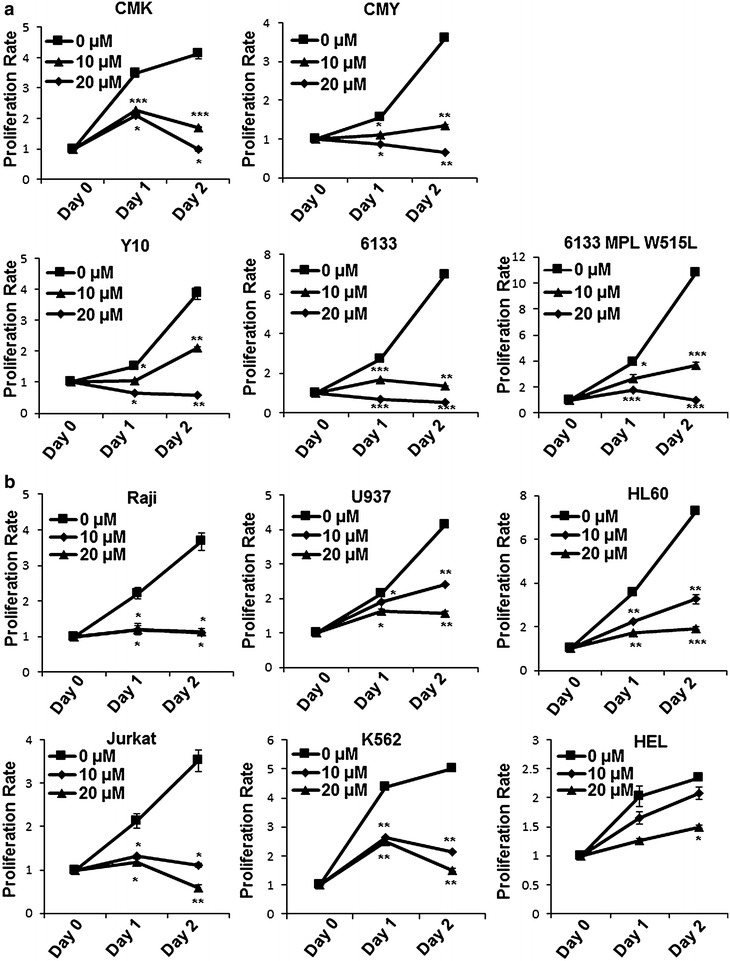
Baicalein inhibited proliferation of leukemia cells. **a** AMKL cell lines (CMK, CMY, Y10, 6133 and 6133 MPL/W515L) and **b** other types of leukemic cells (Raji, U937 HL60, Jurkat and K562) were treated with or without baicalein (0, 10, and 20 µM). The cell numbers of viable cells were determined every 24 h by counting the trypan blue-excluding cells. The cell numbers were normalized to the starting cell number (day 0) and presented as proliferation rate. Data were statistics (Mean ± SD) of a representative experiment (triplicates) from three independent experiments with similar results. *, **, or *** indicates significance (*p* < *0.05*, 0.01, 0.001, respectively) compared to vehicle treatment

To test the effect of baicalein on AMKL cell proliferation, multiple AMKL cell lines including CMK, CMY, Y10, and 6133 were treated with baicalein and the cell proliferation was measured. We found that baicalein efficiently inhibited cell proliferation in a concentration- and time-dependent manner (Fig. 1a). 6133/MPL W515L cells were derived from 6133 with MPL W515L overexpression. These cells proliferated without SCF (stem cell factor) and caused AMKL in mice [27]. Apparently, these cells retained the sensitivity to baicalein treatment similar to 6133 cells (Fig. 1a). We also tested its effect on other types of leukemic cells and observed similar results (Fig. 1b). These observations suggest that baicalein is a potent anti-leukemia reagent. In this study, we focused on AMKL and used 6133 and 6133/MPL W515L cells as models.

### Baicalein induced apoptosis in AMKL cells

**Fig. 2:**
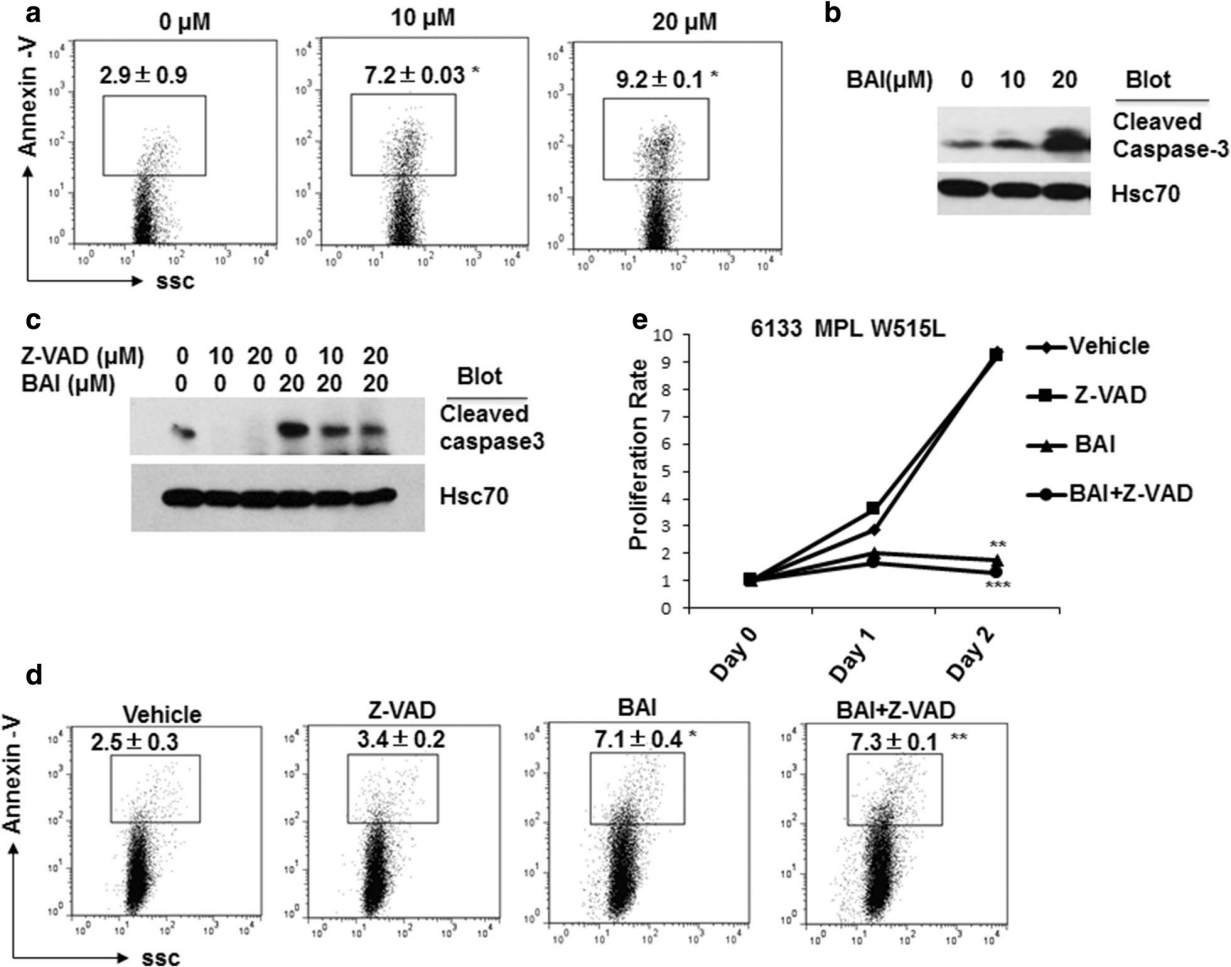
Baicalein induced apoptosis in 6133 MPL/W515Lcells. **a** 6133 MPL/W515L cells were treated with or without baicalein (0, 10, and 20 µM) for 12 h and subjected for Annexin V staining (PE-conjugated antibody) to measure apoptosis. **b** The treated cells were collected for western blot to detect cleaved caspase 3 as an apoptotic marker. Hsc70 serves as a loading control. **c** 6133 MPL/W515L cells were treated with or without baicalein and Z-VAD in combination as indicated for 24 h. The treated cells were collected for western blot to detect cleaved caspase 3 as an apoptotic marker. **d** 6133 MPL/W515L cells were treated with or without baicalein (BAI, 10 µM) and Z-VAD (10 µM) in combination as indicated for 24 h. The treated cells were collected for Annexin V staining to measure apoptosis. **e** The treated cells numbers were counted after 24 h (day 1) and 48 h (day 2) and the proliferation rate was presented. Data were statistics (Mean ± SD) of a representative experiment (triplicates) from three independent experiments with similar results. *p < 0.05, **p < 0.01and ***p < 0.001 as compared to vehicle treatment

To explore how baicalein reduced AMKL cell proliferation, we measured cell death after baicalein treatment. As shown in Fig. 2a, baicalein treatment induced apoptosis evidenced by increased Annexin V staining and the cleavage of caspase 3 (Fig. 2a, b). Although caspase inhibitor Z-VAD reduced the protein level of cleaved caspase 3, Z-VAD treatment did not significantly reduce baicalein-induced apoptosis (BAI vs BAI + z-VAD) (Fig. 2c, d). Accordingly, Z-VAD treatment failed to restore cell proliferation inhibited by baicalein (BAI vs M BAI + Z-VAD) (Fig. 2e). These results suggest that caspase activation may not be the major cause of cell proliferation inhibition by baicalein.

### Baicalein causes cell cycle arrest at G1/G0 phase

**Fig. 3:**
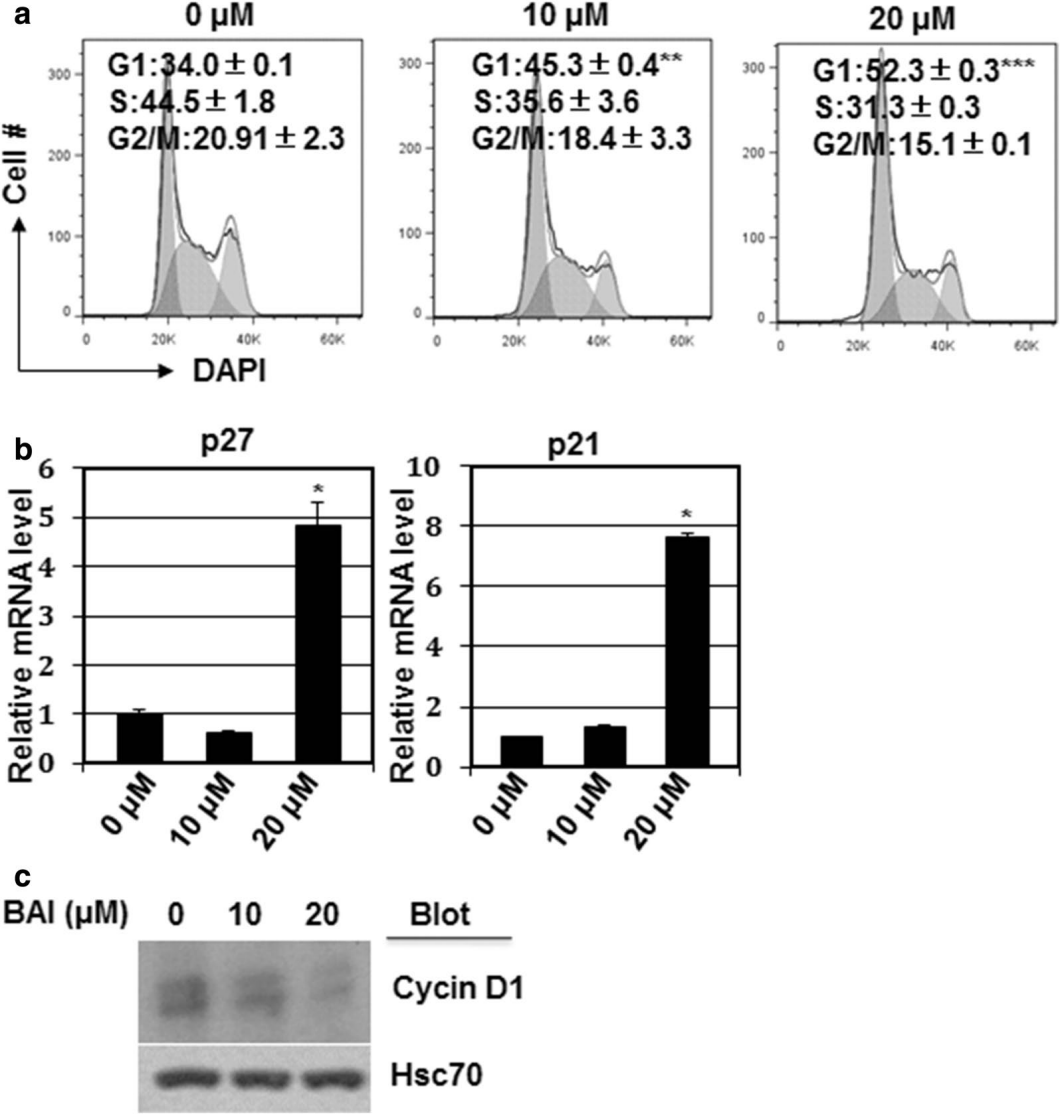
Baicalein caused cell cycle arrest in the G1 phase. **a** Cells were treated with or without baicalein (0, 10, and 20 µM) for 24 h and collected for DAPI staining and FACS analysis. The cell cycle profile was analyzed with a Flowjo software. **b**, **c** The treated cell were collected to measure the mRNA expression level of p21 and p27 and protein expression level of Cyclin D1. *p < 0.05, **p < 0.01 and ***p < 0.001 as compared to vehicle treatment. HSC70 was used as internal control

**Fig. 4:**
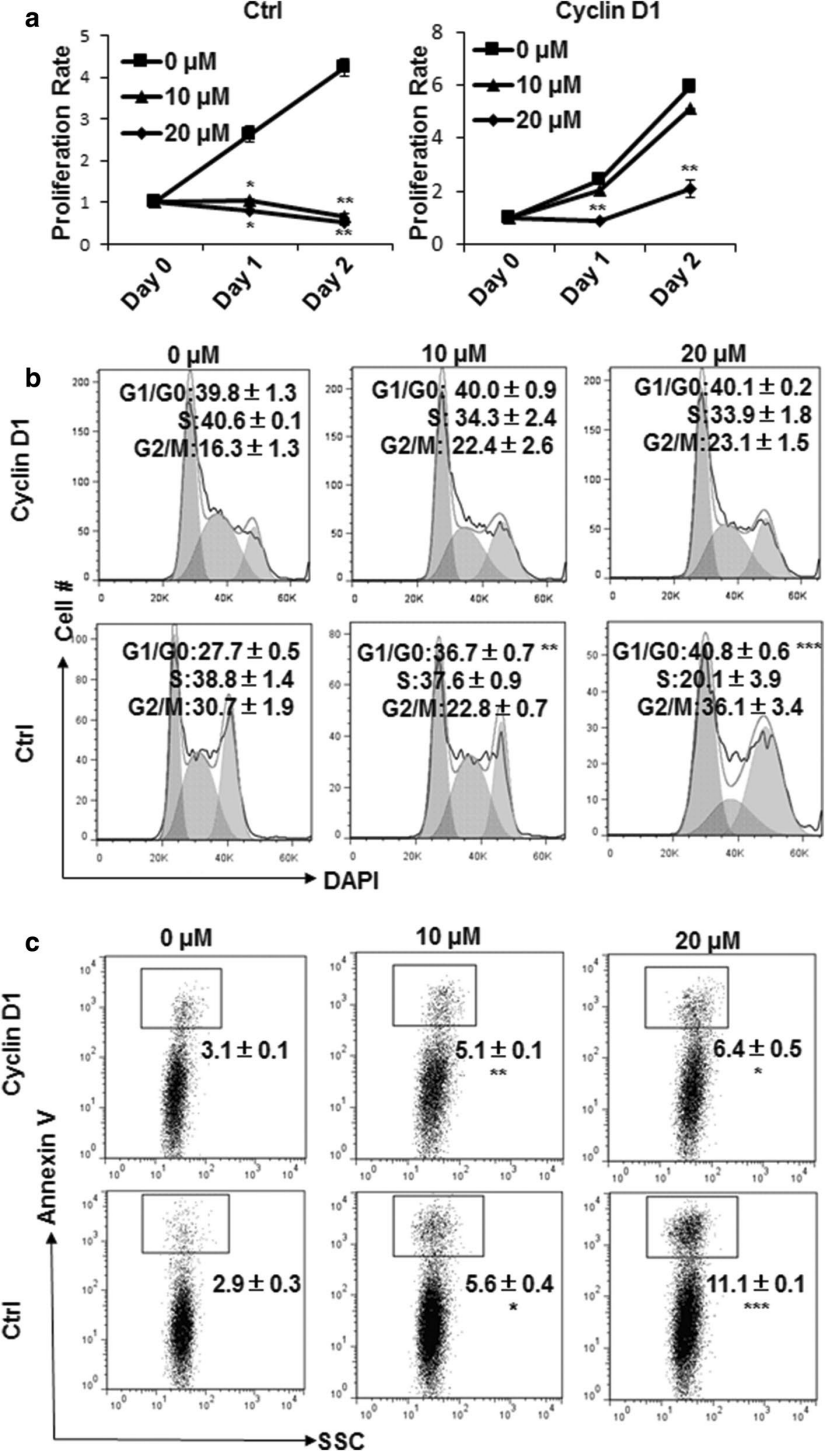
Cyclin D1 overexpression antagonized the effect of baicalein on cell proliferation, apoptosis, and cell cycle. 6133 cells transduced with Cyclin D1 or control vector were treated with or without baicalein (0, 10, and 20 µM). **a** The cell proliferation, **b** cell cycle, and **c** apoptosis were measured as described above. *p < 0.05, **p < 0.01 and ***p < 0.001 as compared to vehicle treatment

To further determine whether baicalein caused cell proliferation inhibition by inducing cell cycle arrest, we first analyzed cell cycle profiles by DAPI staining in 6133 MPL W515L cells treated with baicalein. We found that baicalein treatment significantly reduced the percentage of cells at S phase and increased the percentage of cells at G1 phase (Fig. 3a). Consistent to cell cycle profile, baicalein treatment dramatically elevated the expression of CDK inhibitors p21 and p27 and reduced the expression of Cyclin D1 (Fig. 3b, c). We further tested whether Cyclin D1 overexpression may overcome cell proliferation inhibition by baicalein. As expected Cyclin D1 overexpression promoted cell proliferation. More importantly, Cyclin D1 overexpression efficiently rescued the cell proliferation inhibition, especially at 10 μM concentration (Fig. 4a). In consistent, Cyclin D1 overexpression rescued cell cycle arrest and cell apoptosis induced by baicalein (Fig. 4b, c). These observations suggest that cell cycle arrest may play a major role in baicalein-induced cell proliferation inhibition.

### Baicalein induces AMKL cell differentiation and possesses anti-AMKL potential in vivo

**Fig. 5:**
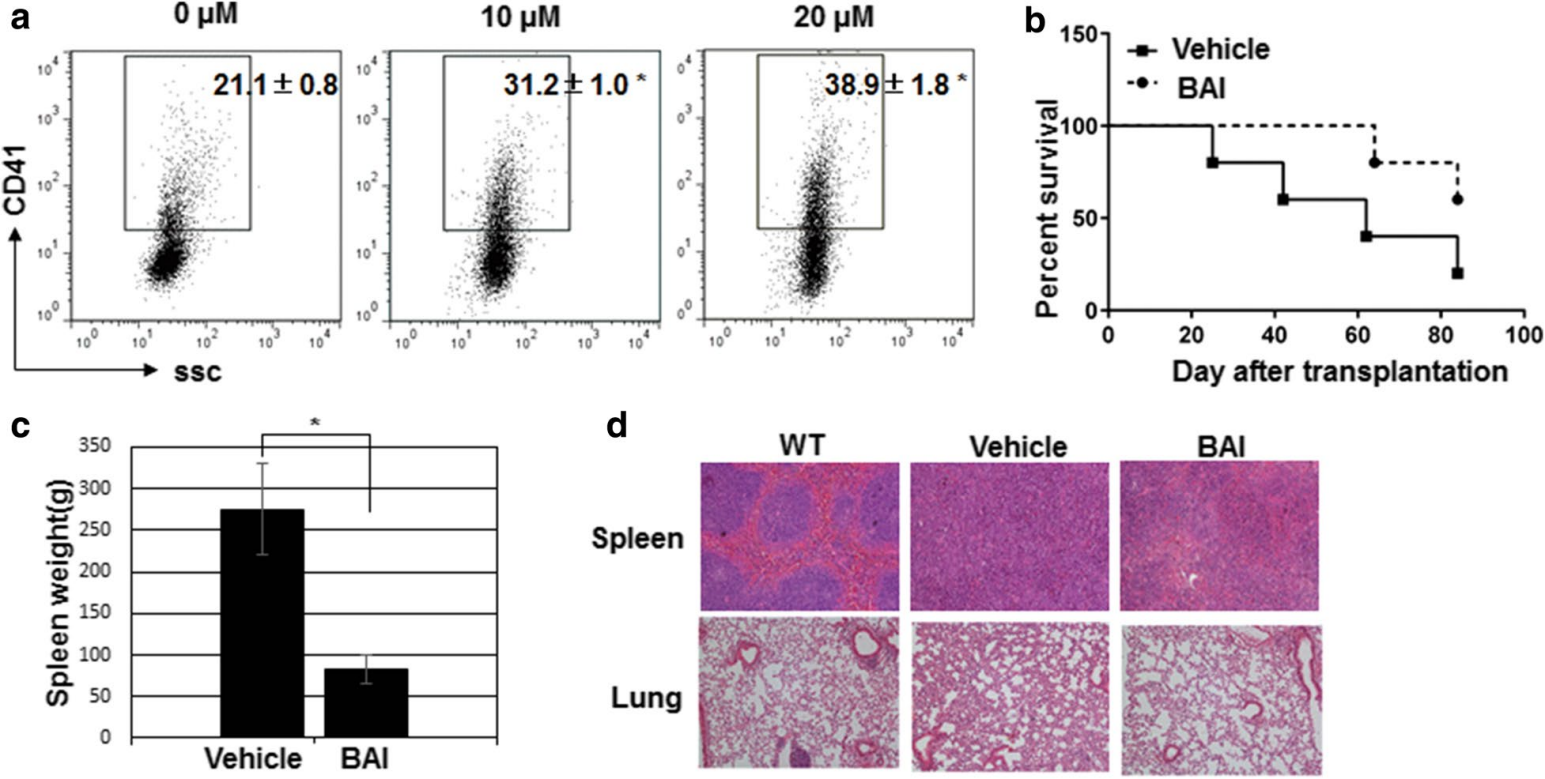
Baicalein possesses anti-AMKL activity in vivo. **a** 6133 MPL/W515L cells treated with or without baicalein were collected for CD41 staining. The expression of CD41 was analyzed by flow cytometry. **b** Sublethally irradiated mice were transplanted with 6133 MPL/W515L cells (3 × 10^5^ cells/mouse). Two days after transplantation, the recepient mice were fed with vehicle or baicalein (20 mg/kg body weight) through oral gavage for 10 days and left for survival observation. **c** The spleen weight from the recipient mice sacrificed on 14 days post transplantation (n = 3, *indicates *p* < 0.05). **d** Histological sections of the spleen and lung from recipient mice sacrificed on 14 days post transplantation were stained with Hematoxylin and eosin (H and E). *p < 0.05 as compared to vehicle treatment

To test whether proliferation inhibition by baicalein was accompanied by cell differentiation, we measured CD41 expression. Baicalein treatment significantly augmented CD41 expression (Fig. 5a). 6133/MPL W515L cells have been shown to induce AMKL in mice and compounds that forced megakaryocyte differentiation possessed potent therapeutic effect on AMKL [8, 9, 13, 27]. To test the effect of baicalein on AMKL in vivo, we transplanted 6133/MPL W515L (carrying GFP for tracing) into sublethally irradiated mice and treated mice with baicalein. 2 days after transplantation, the engraftment was confirmed by monitoring the GFP^+^ in peripheral blood from recipient mice (data not shown). Mice were then randomly divided into two groups for baicalein or vehicle treatment. We found that baicalein treatment significantly promote mice survival (Fig. 5b). Pathology analysis revealed that baicalein significantly reduced blood cell infiltration in spleen and lung (Fig. 5c). In another set of experiment, baicalein also reduced the weight of spleen compared with vehicle treated mice 2 weeks after transplantation (Fig. 5d). These results suggest that baicalein may possess anti-AMKL function.

## Discussion

The prognosis of AMKL is very dismal partially due to failure to identify recurrent genetic alterations for target therapy that urges for development of novel therapeutic methods. Recently, *OTT*-*MAL* and *CBFA2T3*-*GLIS2* have been identified as two recurrent genetic alterations in children type of AMKL and compounds forcing megakaryocyte differentiation possessed potent therapeutic effect in AMKL [8, 9, 13]. In this study, we showed that baicalein possessed anti-AMKL abilities in vitro and in vivo and might be a potent compound for developing novel AMKL therapy.

Although many previous studies have shown the anti-proliferation or anti-tumor functions of baicalein or other similar bioactive components of *Scutellaria* such as wogonin and baicalin in many types of cancer cells, their functions in leukemia cells have not be well characterized. In one study, baicalein inhibited myeloma cell proliferation by suppressing IL-6 signaling [28]. Baicalin induced apoptosis by inducing ROS while wogonin caused apoptosis, inhibited PKC signaling, and inducing differentiation [24, 29]. In our study, we showed baicalein suppressed AMLK cell proliferation by inducing cell cycle arrest and differentiation. Although ROS seemed to be induced by baicalein, the ROS scavenger NAC (N-acetyl-l-cysteine) did not antagonize the inhibitory effect of baicalein on leukemia cell proliferation (data not shown). Considering all these observations, it is possible that baicalein may affect multiple targets and the mechanism by which baicalein exert its function depending on cell context [24, 29–31].

Other mechanism may exist for baicalein to inhibit AMKL cell proliferation. We noticed previous study showing that baicalein activated Notch signaling in K562 cells [22]. Notch signaling was critical for early stage of megakaryocyte commitment and dysregulation of Notch signaling by OTT-MAL caused AMKL through binding to RBPJ, one downstream targets of Notch signaling. These observations suggest Notch signaling may be a common pathway for targeting in leukemia. In our model, the 6133 cells were derived from OTT-MAL knock-in mice developing AMKL. It is possible and worthy to investigate if baicalein may induce megakaryocyte differentiation and inhibit cell proliferation by modulating endogenous Notch signaling in AMKL cells.

## Conclusions

Baicalein induced AMKL cells growth inhibition and apoptosis in vitro, cell cycle arrest may be the key mechanism. Baiclein can relieve disease burden in vivo. Baicalein may be a potent reagent for auxiliary AMKL therapy.

## Methods

### Cell culture and chemical compounds

CMK, CMY, Y10, 6133 MPL/W515L (AMKL cell lines), Raji (human lymphoma cell line), U937 (human macrophage cell line), HL60 (human promyelocytic leukemia cells), Jurkat (acute T cell leukemia), K562 (chronic myelogenous leukmia), and HEL (human erythroleukemia cell) cells were cultured in RPMI-1640 media containing 10 % fetal bovine serum (FBS) and 1 % penicillin–streptomycin (Invitrogen, Carlsbad, CA, USA). 6133 cells were cultured in RPMI-1640 media supplemented with 1 % KIT Ligand conditioned medium as previously described [32]. Baicalein was purchased from Shanxi Ciyuan Biotech Co., Ltd (Xi’an, China). z-VAD-fmk was purchased from EMD Millipore (Billerica, MT, USA). DAPI and NAC (N-acetyl-l-cysteine) were purchased from Sigma (St. Louis, MO, USA).

### Cell proliferation assay

Cells (5 × 10^5^ cells/well) were seeded in a 12-well plate. Drugs were added to the medium at the various concentrations as indicated. After 24 and 48 h incubation, cells were stained with Trypan blue and the Trypan blue-excluded live cells were counted under a light microscopy. The cell numbers were normalized to the starting cell number and presented as proliferation rate. All cell proliferation curves were statistics of one representative experiment (triplicates) from three independent experiments with similar results.

### Cell cycle profiling

Cell cycle profile was assayed by staining with DAPI. Briefly, baicalein-treated cells were fixed, permeablized, and stained with 10 μg/ml DAPI. Data were acquired through Flow Cytometry and the cell cycle profile was analyzed by a FlowJo software (Tree Star,). Flow cytometry was performed on a Calibur (BD Biosciences, Franklin Lakes, NJ, USA) and data were analyzed by a FlowJo software.

### Western blot analysis

Western blot analysis was performed as previously described [32]. Membranes were blotted with antibodies detecting cleaved caspase-3, Cyclin D1, and HSC70. Cleaved caspase-3 and Cyclin D1 antibodies were purchased from Cell Signaling Technologies. HSC70 and horseradish peroxidase-conjugated secondary antibody were purchased from Santa Cruz Biotechnology (Santa Cruz, CA). DAPI and Annexin-V flow antibody were purchased from BD.

### Quantitative RT-PCR

The quantitative RT-PCR was performed as previously described [33]. The PCR primer sets used to detect p21 and p27 were as following:

p21: sense 5-ACCAGCCTGACAGATTTCTA-3 and antisense 5-TGACCCACAGCAGAAGAG-3; P27: sense 5-AGTGTCCAGGGATGAGGA-3 and antisense 5-GGGAACCGTCTGAAACAT-3. The internal control using the primers were: GAPDH: sense 5-GGTGAAGGTCGGTGTGAACG-3 and antisense 5-CTCGCTCCTGGAAGATGGTG-3.

### Retroviral transduction

For Cyclin D1 overexpression, retrovirus (pMIGR1) was used. The retroviral stocks were prepared as previously described and used for infection of 6133 cells [32]. We obtain 6133 cells with stable expression of Cyclin D1 by flow cytometry sorting GFP + cells.

### AMKL mouse model

AMKL mouse model was established as previously described [13]. Briefly, recipient mice were sublethally (600 rad) irradiated and 6133/MPL W515L cells (3 × 10^5^) were introduced into recipient mice through tail vein injection. Two days after injection, mice were randomly divided into control and experiment groups and received vehicle or baicalein (20 mg/kg body weight) through oral gavage for 10 days. Mice were sacrificed on day 14 for disease burden analysis or maintained to end point for survival rate analysis. Log-rank (Mantel-Cox) test was performed to measure the statistic significance of the difference between vehicle and baicalein treatment groups. All procedures involving animals were approved by the Animal Care and Use Committee of Wuhan University.

### Statistical analysis

Student’s *t*-test (unpaired, two-tail) was used for statistical analysis and a *p* value <0.05 was considered as significance.

## Abbreviations

AMKL: acute megakaryoblastic leukemia
SCF: stem cell factor
NAC: N-acetyl-l-cysteine
ROS: reactive oxygen species
DCFH‑DA: 2′, 7′‑dihydrodichlorofluorescein diacetate
FACS: fluorescence activated cell sorter
DAPI: 4′, 6-diamidino-2-phenylindole
GAPDH: glyceraldehyde 3-phophate
HSC70: heat Shock Cognate Protein 70
